# Total Internal Reflection Fluorescence Quantification of Receptor Pharmacology

**DOI:** 10.3390/bios5020223

**Published:** 2015-04-27

**Authors:** Ye Fang

**Affiliations:** Biochemical Technologies, Science and Technology Division, Corning Incorporated, Corning, NY 14831, USA; E-Mail: fangy2@corning.com; Tel.: +1-607-974-7203; Fax: +1-607-974-5957

**Keywords:** evanescent wave, receptor pharmacology, resonant waveguide grating, single molecule analysis, surface plasmon resonance, total internal reflection fluorescence

## Abstract

Total internal reflection fluorescence (TIRF) microscopy has been widely used as a single molecule imaging technique to study various fundamental aspects of cell biology, owing to its ability to selectively excite a very thin fluorescent volume immediately above the substrate on which the cells are grown. However, TIRF microscopy has found little use in high content screening due to its complexity in instrumental setup and experimental procedures. Inspired by the recent demonstration of label-free evanescent wave biosensors for cell phenotypic profiling and drug screening with high throughput, we had hypothesized and demonstrated that TIRF imaging is also amenable to receptor pharmacology profiling. This paper reviews key considerations and recent applications of TIRF imaging for pharmacology profiling.

## 1. Introduction

The evanescent field is a near-field wave that decays in intensity over a sub-wavelength distance, and has been used as the basis for developing several near-field fluorescent imaging techniques including total internal reflection fluorescence microscopy (TIRFM) [1], near-field scanning optical microscopy (NSOM) [2], and virtual supercritical angle fluorescence microscopy [3]. Among them, TIRFM uses an evanescent wave generated at a dielectric interface with an incident angle greater than the critical angle to selectively excite and visualize local fluorophores. The critical angle is the angle of incidence above which total internal reflection (TIR) occurs. TIRFM has become one of the most powerful techniques to quantify the dynamics and kinetics of single molecule reactions *in vitro* and in living cells [4,5,6]. However, TIRFM has been mostly used as a low throughput imaging tool and has found little use in high content screening and receptor pharmacology profiling, due to its complexity in instrumental setup and experimental procedures [7,8].

In recent years, label-free evanescent wave biosensors including resonant waveguide grating (RWG) and surface plasmon resonance (SPR) have found applications in both cell biology [9,10,11,12,13,14] and cell phenotype-based drug discovery processes [15,16,17,18,19]. Similar to TIRFM, these biosensors also employ a surface-bound evanescent wave to noninvasively monitor in real time a whole-cell response, termed dynamic mass redistribution (DMR) signal, of living cells upon stimulation [10,11]. In particular, by using microplate formats that are *de facto* footprints for drug discovery, RWG biosensor permits cell phenotypic profiling and high throughput screening of drugs in native cells [15,16,17,18,19]. These cell phenotypic assays have gained increasing acceptance in both basic research [20,21] and early drug discovery processes [22,23]. Inspired by the success of these biosensors, we had hypothesized and demonstrated recently that TIRFM, in particular a microplate-compatible TIRF instrument, can be used to characterize receptor pharmacology [24,25].

This paper reviews theories, instrumentation, and primary applications in cell biology of several types of TIRFM, and discusses critical considerations and the development of TIRFM for pharmacology profiling.

## 2. Evanescent Wave

**Figure 1:**
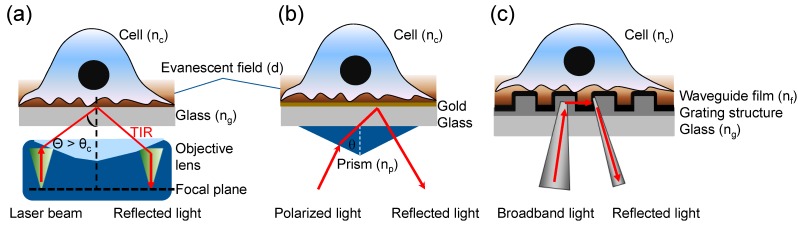
Three types of evanescent wave-excited fluorescence microscopy. (**a**) Through-the-objective TIRFM, wherein a high numerical aperture (NA) objective lens is used to simultaneously generate the evanescent field at the cell-glass interface and view the cell. A laser light is directed and focused on the back focal plane, which then creates a refracted parallel beam approaching the interface in the small gap between objective and glass coverslip. TIR is achieved when the angle is greater than the critical angle (θ > θ_c_); (**b**) Prism-based surface plasmon-excited TIRFM, wherein an incident light is directed onto a gold film via a prism, creating an electromagnetic field penetrating into the cell under resonance condition. The reflected beam is detected via a photodetector or imager for SPR measurement, while the excited fluorescence is collected using a separate objective; (**c**) Resonant waveguide grating-based TIRFM, wherein a grating is used to couple light into the waveguide, creating an evanescent field resulting from total internal reflection of the light beam. The excited fluorescence is collected using a CCD camera.

TIRFM uses an evanescent electromagnetic field to selectively excite and visualize fluorescent molecules in the close vicinity of a substrate. TIRFM generally uses three distinct configurations: glass coverslip/sample, glass/gold film/sample (SPR), and glass/grating waveguide film/sample (RWG) (Figure 1). For cell biology applications the sample is adherent cells in aqueous solution.

For the glass coverslip/sample configuration that is most commonly used, the TIR is based on Snell’s law:

(1) $$n_{g}\sin\theta_{1}=n_{c}\sin\theta_{2}$$

where θ_1_ is the angle of incidence, θ_2_ the angle of refraction, and *n_g_* and *n_c_* the refractive indices of the coverslip and the sample, respectively. To achieve TIR the refractive index of the sample must be less than that of the coverslip. The Snell’s law describes that light will undergo TIR, if the light travelling in a dense medium with a high refractive index (RI) (*n_g_*) strikes a less dense medium with a low refractive index (*n_c_*) beyond a certain critical angle (θ*_c_*). At the critical angle θ*_c_*, θ_2_ equals to 90°. Thus, the critical angle is:

(2) $$\theta_{c}=\sin^{-1}(n_{c}/n_{g})$$

For typical TIRF setup, the glass of a cover slip has a RI of 1.515 (*n_g_*), while an adherent cell has a RI of 1.36 (*n_c_*). Thus, the critical angle is 63.85° [26] (Figure 1a).

Under TIR condition an evanescent field is created at the dielectric interface and extended into the sample. The intensity, *I_z_*, of the evanescent field at any position *z* is:

(3) $$I_{z}=I_{0}e^{-z/d_{p}}$$

where *I*_0_ is the intensity of the evanescent field at *z* = 0, and *d* is the penetration depth. *I*_0_ is related to the intensity of the incident beam by a complex function of incident angle and polarization. The penetration depth is the distance from the coverslip at which the excitation intensity decays to 1/e, or 37%, of *I*_0_. The penetration depth into the sample media, *d_p_*, is [27]:

(4) $$d_{p}=\frac{\lambda }{4\pi \sqrt{n_{g}^{2}\sin^{2}\theta_{1}-n_{c}^{2}}}=\frac{\lambda }{4\pi n_{g}^{2}\sqrt{\sin^{2}\theta_{1}-\sin^{2}\theta_{c}}}$$

where λ is the vacuum wavelength of incident light. The penetration depth depends on incidence angle, wavelength, and refractive indices of the sample and the coverslip. However, only the incidence angle is practically varied for a given instrument setup. The depth decreases as the incident angle increases, and generally ranges from about λ at θ just slightly greater than θ*_c_*, down to about λ/10 at supercritical θ*_c_* [27]. Typical depths are in the range 60–100 nm.

SPR uses an electrically conducting gold film to convert the incident light photons into surface plasmons (SPs) (Figure 1b). In a Kretschmann configuration the TIR occurs when the magnitude of the parallel wave vector of the evanescent wave, κ*_x_*, is the same as the magnitude of the wave vector of the surface plasmon, κ*_sp_*:

(5) $$\begin{array}{l} k_{sp}=\frac{2\pi }{\lambda }\sqrt{\frac{n_{c}^{2}n_{g}^{2}}{n_{c}^{2}+n_{g}^{2}}}\\ =k_{x}=\frac{2\pi }{\lambda }n_{p}\sin\theta \end{array}$$

where λ is the wavelength of incident light, *n_c_* is the refractive index of sample, *n_g_* is the refractive index of gold, *n_p_* is the refractive index of prism, and θ is the incident angle. The resonant or critical angle, θ*_c_*, is [28]:

(6) $$\theta_{c}=\sin^{-1}\left(\frac{1}{n_{p}}\sqrt{\frac{n_{c}^{2}n_{g}^{2}}{n_{c}^{2}+n_{g}^{2}}}\right)$$

The SPR angle is about 35.21° in air and increases linearly with the increasing RI of the solution at the interface. The gold film is at the interface of a glass substrate and a sample (e.g., cells or aqueous medium), and the plasmons create an evanescent wave field that extends into the medium on either side of the gold film. The penetration depth into the sample medium, *d_SPR_*, is [29,30]:

(7) dSPR=Im[λ4πnc(εm+nc2)1/2]

where ε*_m_* is the dielectric constant of gold. Theoretical [29,30] and experimental data [31] suggest that the penetration depth at resonance grows non-linearly from ~100 nm to 5 µm as the wavelength increases from 600 nm to 4 µm.

RWG use a diffractive nanograting waveguide structure to create an evanescent wave under resonant coupling (Figure 1c). In a typical three-layer RWG sensor the coupling of light into the waveguide through diffraction is governed by:

(8) $$n_{2}\sin\theta_{m}=n_{i}\sin\theta_{in}+\frac{m\lambda }{\Lambda }$$

where *n_i_* and *n*_2_ is the refractive index before and after the grating interface, θ*_in_* the angle of incidence of the incoming light, θ*_m_* the propagation angle of the diffracted order *m*, *m* = 0, ±1, ±2, …, λ the wavelength of the incident light, and Λ the grating period. For RWG, the grating is typically a sub-wavelength structure with a period of the grating smaller than the incident wavelength. Under such condition only the zeroth order diffraction exists, for which the grating equation is reduced to classical Snell’s law. For the leaky waveguide modes inside RWG structures using a transverse magnetic (TM) polarized incident light, the angle of incidence can be quite small. For Epic^®^ (Corning Incorporated, Corning, NY, USA) biosensors the resonant angle is close to normal incident angle when the incident wavelength of ~830 nm is used [32]. The penetration depth of a given mode is [33]:

(9) $$d_{RWG}=\frac{1-\sigma }{k\sqrt{N^{2}-n_{c}^{2}}}+\frac{\sigma {\left[{\left(\frac{N}{n_{f}}\right)}^{2}+{\left(\frac{N}{n_{c}}\right)}^{2}-1\right]}^{-1}}{k\sqrt{N^{2}-n_{c}^{2}}}$$

where *n_c_* is the refractive index of sample, *n_f_* the refractive index of the waveguide film, *N* the effective index of each mode, κ the wave vector (2π/λ), and σ is 0 for transverse electric (TE) mode and 1 for TM mode. The TM_0_ mode is the most commonly used for sensing due to its highest sensitivity [11].

## 3. TIRFM Instrument Configurations

**Figure 2:**
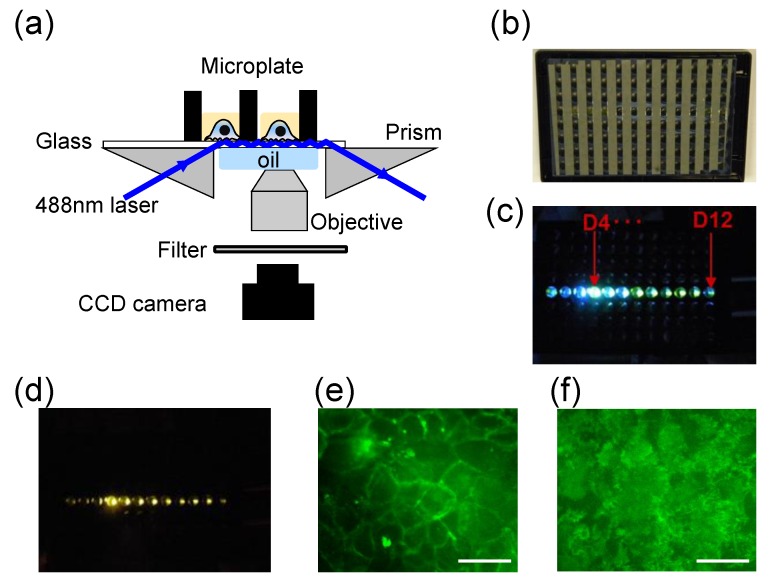
Microplate-compatible TIRF imaging system. (**a**) Instrument setup. A 488 nm laser light is directed to illuminate the glass substrate after it has been guided through a right angle prism. Under TIR condition the evanescent wave excited fluorescence is collected using an objective lens, passed through a filter and focused via a tube lens onto a CCD camera; (**b**) The back image of a glass bottom microplate to show the geometry of alumina stripes; (**c**) The excitation light propagates within the glass substrate after it illuminates the well D4; (**d**) Whole plate TIRF observed within multi-wells by placing a filter above the CCD camera; (**e**) Epi-fluorescence image of HEK-β_2_AR-GFP cells; (**f**) TIRF image of the cells. Scale bar in (**e**,**f**) is 40 µm. This figure was reproduced with permission from [25].

Most TIRFM systems use laser beams as incident light. However, TIRFM varies greatly in instrument setup, which generally falls into: through-the-objective, through-the-prism, RWG-based configurations (Figure 1), and around-the-objective (Figure 2a). Currently, most TIRF microscopes are objective-based.

Through-the-objective TIRFM (oTIRFM) uses a high numerical aperture (NA) objective to simultaneously refract incident light beyond critical angle for illumination and collect fluorescence emission (Figure 1a). The NA of an objective describes its light-gathering power—specifically, the angle through which the objective is able to collect light, or the maximum angle at which the excitation light can emerge from the objective. For cell imaging, a prerequisite is that the NA is greater than the RI value of living cells (*n_c_* = 1.37). The higher NA allows larger incidence angles. For oTIRFM a laser is directed and focused on the back focal plane of the objective so a refracted parallel beam can leave the front optical plane of the objective and impinge on the coverslip/aqueous interface at angles beyond θ*_c_* [34]. The objective collects both propagating and evanescent emission due to the proximity of the TIR interface to the power-normalized emitting dipole [35]. Evanescent emission intensity depends on the dipole-to-interface distance and is a substantial portion of the total emitted power for a dipole on the interface. oTIRFM has high light collection efficiency and high spatial resolution, and requires minimal maintenance and alignment. oTIRFM is also convenient as the specimen is easily accessible and the incidence angle can be changed easily. However, oTIRFM has relatively high background autofluorescence from the glass elements of the objective [36,37].

Prism-based TIRFM (pTIRFM) uses a prism to direct light into a cover glass or a SPR sensor chip (Figure 1b). The evanescent field-excited fluorescence is collected from the aqueous side with a water immersion objective lens or from the glass side with oil immersion objective. Owing to separated paths for excitation and emission pTIRFM produces an evanescent field with less scattered light, lower background and a greater range of incidence angles, compared to oTIRFM [38]. Coupled with a wide laser beam pTIRFM permits a wide field of view. However, pTIRFM does not collect near-field emission from the fluorescent sample and has restricted sample accessibility when images are collected from the aqueous side.

RWG-based TIRFM (rTIRFM) uses a subwavelength diffractive nanograting to couple light into, and travel along, a thin film dielectric transparent waveguide, so an evanescent wave is generated to excite near-field fluorescence (Figure 1c) [24,39,40]. The resonance effect occurs when the grating couples incident light to the specific modes of the waveguide. The RWG functions as a very narrow band-stop filter, since the portion of transmitted light is close to zero due to the destructive phases of the transmitted and outcoupled light at the backside of the grating [41]. The evanescent wave properties can be tuned by altering the grating parameters and RI difference between the waveguide and the surrounding substances; for instance, its penetration depth can be varied, which is important for cell analysis [42]. Under resonance condition the incoupled light is concentrated into a shallow waveguide structure, leading to a high intensity electromagnetic field inside it, which can be used to greatly enhance the excitation of fluorescent molecules at the interface [43,44].

Around-the-objective TIRFM (aoTIRFM) uses a laser beam to propagate through the submillimeter gap created by an oil immersion high NA objective and the glass coverslip (Figure 2a) [45]. This approach collects both propagating and evanescent emission, and eliminates background light arising from the admission of the laser excitation to the microscopic optics. Both upright and inverted microscopic configurations can be realized. In an inverted configuration, the exciting beam is propagated into the immersion oil with an additional reflection of the beam at a glass/air interface as the beam approaches or retreats from the objective. Notably, aoTIRF can produce an evanescent field with ultrashort penetration depth (~50 nm) [45].

Current TIRFM exclusively deals with single samples, one measurement at a time. However, to meet the demand in high throughput for pharmacology profiling and screening, there are several additional factors to be considered. First, TIRFM should be applicable to microplates, the *de facto* format for high throughput profiling and screening. Second, TIRF excitation should be scalable to multiple wells (e.g., a single column or row within a microplate) simultaneously, which is important to simplify instrument setup. Third, TIRF excitation and imaging have to be done from the bottom of a microplate, because of the requirement for sample handling and compound addition. Fourth, TIRF excitation should be consistent across the microplate, given that profiling often requires comparative analysis of different compounds or a compound at different doses. On the other hand, compared to single molecule applications, spatial resolution is less demanding for profiling. This is because profiling generally uses an averaged response of a relatively large number of cells for comparison.

We recently had developed a prism-based and microplate-compatible TIRF imaging system to speed up TIRF imaging [25]. This system is similar to an aoTIRFM setup (Figure 2a). Here, a 488 nm diode laser is guided through a right-angle prism to launch a light beam into bottom glass substrate of a well located at the left side of a microplate. The microplate has an array of 8 × 12 wells with an aluminum thin film strip separating each column on the backside of the glass bottom (Figure 2b). The aluminum strips are perpendicular to the light propagation direction under TIR condition, and are used to reduce the evanescent wave attenuation and to minimize the leakage of the TIR light into the adhesives between glass sheet and the bottom of the holey microplate (Figure 2c). This is essential to simultaneously illuminate multiple wells along the propagation direction. When a laser light is launched through a prism attached to the left side of the microplate (e.g., the well D4) and the incident angle is greater the critical angle, the excitation light was found to undergo TIR and propagate within the glass plates all the way to the well D12 (Figure 2d). Immersion oil is used to fill the gap between the prism and the glass bottom. A CCD camera, together with a 50× objective and a tube lens with a desired effective focal length, are used to record TIRF images. By placing a band pass filter above the CCD camera, TIRF emission from multi-wells can be observed coincidentally (Figure 2d). This system can perform both epi-fluorescence and TIRF imaging, depending on the laser incident angle (Figure 2e,f, respectively).

## 4. Applications of TIRFM in Cell Biology

**Figure 3:**
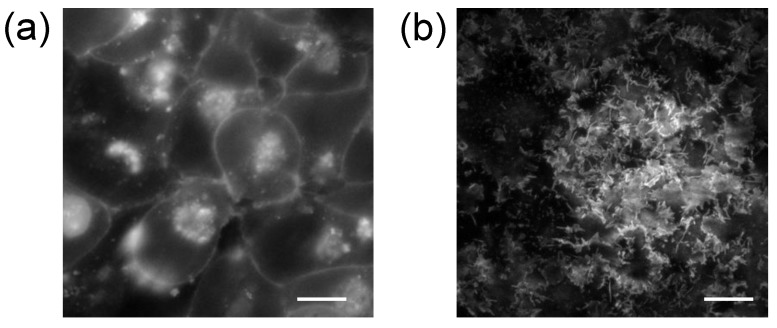
Comparison of epi-fluorescence (**a**) and TIRF (**b**) images of the same HEK-β_2_AR-GFP cells cultured on glass coverslip. These cells express green fluorescent protein (GFP) tagged β_2_-adrenergic receptor (β_2_AR), a prototypical G protein-coupled receptor. Scale bar is 10 µm. In the TIRF image, only GFP-tagged receptors at the cell membrane closest to the cover slip are visible. Furthermore, TIR illumination allows more single molecules to be detected and results in brighter fluorescence than epi-illumination.

Since the evanescent field intensity decays exponentially with distance from the interface, TIRFM gives rise to a very narrow excitation depth, suggesting that only a small section at the base of adherent cell is excited. Naturally occurring fluorophores in the cell bulk, such as NADH and flavins, are often out of focus, leading to very high signal-to-background ratios (SBRs). Compared to epi-fluorescence imaging in which both excitation and emission typically involve freely propagating light, TIRFM is unique in that it only produces surface-selective images (Figure 3). The ability to perform such an optical sectioning makes TIRFM the method of choice to visualize single molecules in living cells, in particular fluorescent molecules located at the cell adhesion sites, and in cell membrane and membrane proximal cytoplasmic organelles. TIRFM has been widely used to study dynamic events close to the plasma membrane of living cells, including cell adhesion, endocytosis, exocytosis, ligand binding to cell surface receptor, cytoskeletal remodeling, dynamic membrane microdomains, and forward trafficking (*i.e.*, targeting of intracellular proteins to cell membrane) (reviewed in [4,5,46,47,48]). This section reviews recent progress of TIRF techniques for cell analysis.

TIRFM is mostly restricted to a single plane and commonly viewed as a two-dimensional (2D) imaging tool for surfaces or structures extremely close to surfaces [49]. The shape of the exponential decay of the evanescent field is known to contain information about the axial position of a fluorophore or a fluorescent feature of interest [50]. Furthermore, since the penetration depth of the evanescent wave decreases as the incidence angle increases, it is possible to use multiple incidence angles to provide a third dimension for TIRFM imaging [51]. However, these approaches still remain in the realm of localization techniques. To overcome this limitation, Boulanger *et al.* developed a novel TIRF imaging modality based on a versatile design enabling fast multi-wavelength azimuthal averaging and incidence angles scanning [52]. This three-dimensional (3D) reconstruction approach uses multi-angle TIRFM image stacks for investigating dynamic processes occurring in depth of the cell up to 800 nm from the plasma membrane. Specifically, they demonstrated that it is possible to obtain structural and dynamical information about 3D actin architectures in living cells, and temporally elucidate two distinct recycling transport intermediates in Rab11a-dependent exocytosis.

Since the evanescent field intensity is several times stronger than the incident intensity for incident beam angles within about 10° of the critical angle, TIRF gives improved signal-to-noise ratio (SNR) [53]. The use of SPR [54] or RWG [41,44] can result in up to several hundred-fold enhancement of fluorescence, further increasing the SNR. Combined with high SBR, TIRFM permits single molecule detection on the basolateral surface of cells [4,5]. Single molecule tracking is useful to determine the patterns of interaction between molecular species and the kinetic rates of association and dissociation from the position of interacting molecules *vs.* time. G protein-coupled receptors (GPCRs) constitute the largest family of receptors and a major class of drug targets in the human genome [55]. Many GPCRs are known to form constitutive dimers or higher oligomers; however, the size and stability of such complexes under physiological conditions are largely unknown [56]. Hern *et al.* used Cy3B-telenzepine, an antagonist, to label M_1_ muscarinic acetylcholine receptor on CHO cells, and then tracked the position of individual receptors over time using TIRFM [57]. They found that in isolated CHO cells receptors are randomly distributed over the plasma membrane, and ~30% of the receptor molecules exist as dimers at any given time, and receptors can undergo interconversion between monomers and dimers on the timescale of seconds. In another study, Calebiro *et al.* used TIRFM to track single receptors having a SNAP tag, and compare the spatial arrangement, mobility, and supramolecular organization of three prototypical GPCRs: β_1_-adrenergic receptor (β_1_-AR), β_2_-AR, and γ-aminobutyric acid (GABAB) receptor [58]. Results showed that these GPCRs showed very different degrees of di-/oligomerization, the lowest for β_1_ARs (monomers/dimers) and highest for GABAB receptors (prevalently dimers/tetramers of heterodimers). Whereas β_1_- or β_2_-ARs were apparently freely diffusive on the cell surface, GABAB receptors were prevalently organized into ordered arrays via interaction with the actin cytoskeleton. Agonist stimulation did not alter receptor di-/oligomerization, but increased the mobility of GABAB receptor complexes. These results suggest that GPCRs are present on the cell surface in a dynamic equilibrium, with constant formation and dissociation of new receptor complexes that can be targeted, in a ligand-regulated manner, to different cell-surface microdomains.

Single molecule tracking is also useful to study the forward trafficking or membrane targeting of intracellular proteins. For instance, single-molecule TIRFM imaging revealed that PTEN, a tumor suppressor, binds to the cell membrane for a few hundred milliseconds, sufficient to degrade several phosphatidylinositol-3,4,5 trisphosphate (PIP3) molecules [59]. Interestingly, the steady-state level of bound PTEN is the highest at sites of retracting membrane, including the rear of highly polarized cells. Recently, McGuire *et al.* had developed a fully automated single molecule fluorescence counting method that separates tagged proteins on the plasma membrane from background fluorescence and contaminant proteins in the cytosol or the endoplasmic reticulum and is useful to determine protein stoichiometry [60].

TIRFM has been widely used for studying cell adhesion dynamics. However, challenges in image capture and downstream analysis have generally limited its use to characterize a relatively small number of hand-picked adhesions within any given cell. Recently, Berginski *et al.* described a 60× oil-immersion TIRF system for the quantification of focal adhesion dynamics using an analysis system that identifies individual adhesions labeled with EGFP-Paxillin in migrating NIH 3T3 fibroblasts based on user-defined criteria, leading to high-resolution data sets of adhesion distribution, morphology, and turnover [61]. Focal adhesions are dynamic, multi-component protein complexes that serve as points of integration for both mechanical and chemical signaling, while playing a central role in a variety of processes including cancer metastasis, atherosclerosis and wound healing. Their results showed that a single point mutation in paxillin at the Jun-kinase phosphorylation site Serine-178 can significantly change the size, distribution, and rate of assembly of focal adhesion complexes.

The evanescent field has polarization components in each of the three spatial axes. Specifically, when the incident light is *p*-polarized in the x–z plane, the corresponding *s*-polarization is parallel with the y-axis. Alternatively, *p*-polarization can be in the y–z plane and *s*-polarization parallel with the x-axis. The fluorescence intensity from the molecule is known to depend on its orientation and the polarization of the evanescent field. Thus, switching the polarization of the incident light between the *s*- (perpendicular to the plane of incidence and parallel to the coverslip) and *p*-directions (in the plane of incidence and perpendicular to the coverslip) alternates the evanescent field polarization between y- and z-directions, yielding a polarization-dependent fluorescence intensity from the molecule, which can be used to determine the orientation of its excitation dipole [62]. This approach uses both p- and s- evanescent field polarizations to sequentially excite a membrane-embedded probe such as carbocyanine dye diI-C18-(3) (DiI). DiI has been shown to embed in the membrane with its transition dipole moments nearly in the plane of the membrane. Regions, even submicroscopic ones, in which the membrane deviates from parallelism with the substrate, are vividly highlighted by taking the ratio p-pol/s-pol (P/S) of the membrane-embedded diI fluorescence images [63,64,65,66,67]. In living cells, variations in membrane orientation occur in many cellular processes, including endocytosis, exocytosis, cell surface ruffling, and receptor signaling.

## 5. TIRFM for Pharmacology Profiling

**Figure 4:**
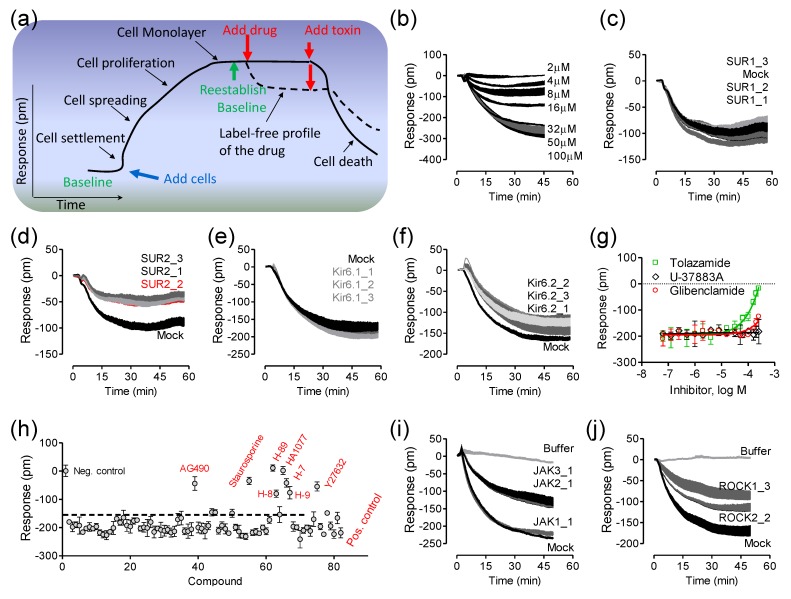
Label-free cell phenotypic assays for decoding receptor composition and signaling. (**a**) A hypothetical kinetic response of living cells on the sensor surface; (**b**) The real-time DMR dose responses of pinacidil in HepG2C3A cells; (**c**–**f**) The DMR of 40 µM pinacidil in the mock- transfected cells, in comparison with those in cells treated with three different SUR1 RNAi (**c**); SUR2 RNAi (**d**); Kir6.1 RNAi (**e**); or Kir6.2 RNAi (**f**); (**g**) The DMR of 32 µM pinacidil as a function of different K_ATP_ blockers; (**h**) The sensitivity of 32 µM pinacidil DMR to kinase inhibition, plotted as its amplitudes at 50 min post stimulation as a function of compound; (**i**) The real-time DMR of 40 µM pinacidil with mock transfection (mock) or JAK siRNA; (**j**) The real-time DMR of 40 µM pinacidil with mock transfection (mock) or ROCK siRNA. For (**c**–**f**) and (**i**–**j**), RNAi transfection was done 48hrs prior to the pinacidil stimulation. For (**g**,**h**), the cells were first treated with each blocker for 1hr, followed by stimulation with 32 µM pinacidil. For (**i**) and (**j**), the buffer DMR was included as a negative control. Data represents mean ± s.d. (*n* = 6 for **a**–**f**, *n* = 3 for g, *n* = 4 for h, *n* = 6 for **i** and **j**). This figure is reproduced from Ref. [14] through the Creative Commons Attribution License.

In recent years, label-free optical biosensors such as RWG and SPR have been used to study many fundamental aspects of cell biology including cell adhesion, cycle, proliferation, receptor signaling, death, cell barrier functions, cell-to-cell communication, migration, invasion, differentiation, and viral infection (reviewed in [68,69,70,71,72,73,74,75]). This is due to the ability of these biosensors to non-invasively monitor the entire life cycle of living cells on the sensor surface (Figure 4a). These biosensors offer great flexibility in assay formats, so a wide range of cell phenotypes can be studied [19,71,73]. In particular, the RWG biosensor has been found to be a powerful high-throughput technique for receptor biology studies [10,11,12,13,14,20,21] and drug profiling and screening [15,16,17,18,19,22,75,76].

This is exemplified by the deconvolution of the composition and signaling of an endogenous ATP-sensitive potassium channel (K_ATP_) in HepG2C3A cells [14]. Here, cells are first cultured directly in a 384 well microplate, each well containing a single RWG biosensor, until the cells form a confluent monolayer. After re-establishing a baseline, a drug compound is added into each well and the cellular response is monitored in real-time, resulting in a label-free profile, also termed as label-free cell phenotypic profile, for the drug molecule (Figure 4a). DMR agonist profiling of a library of seventy-two ion channel ligands, each at 10 µM, in five distinct cell lines showed that pinacidil, a K_ATP_ channel opener, triggered a robust negative DMR in A431, A549, HT29 and HepG2C3A, but not HepG2 cells. Pinacidil was later found to result in a dose-dependent response with a logEC_50_ of −4.77 ± 0.05 (*n* = 6) in HepG2C3A cells (Figure 4b). RNAi knockdown of known K_ATP_ channel component proteins was then used to decode the ion channel composition (Figure 4c–f). Results showed that only RNAi knockdown of SUR2 greatly suppressed the pinacidil DMR (Figure 4d), suggesting that the pinacidil activated channel is SUR2-containing K_ATP_ channel. Consistent with RNAi knockdown results is that tolazamide blocked the pinacidil DMR, while glibenclamide up to 250 µM only partially attenuated the pinacidil DMR, and U-37883A had little impact on the pinacidil DMR (Figure 4g). Tolazamide is a sulfonylurea blocker, glibenclamide is a relatively selective SUR1 inhibitor, but U-37883A is a non-sulfonylurea blocker that selectively inhibits Kir6.1 containing KATP channels. Kinase inhibition (Figure 4h) and RNAi knockdown (Figure 4i,j) further showed that the pinacidil activated K_ATP_ channels trigger signaling through Rho kinase (ROCK) and Janus kinase-2 (JAK2) and JAK3.

**Figure 5:**
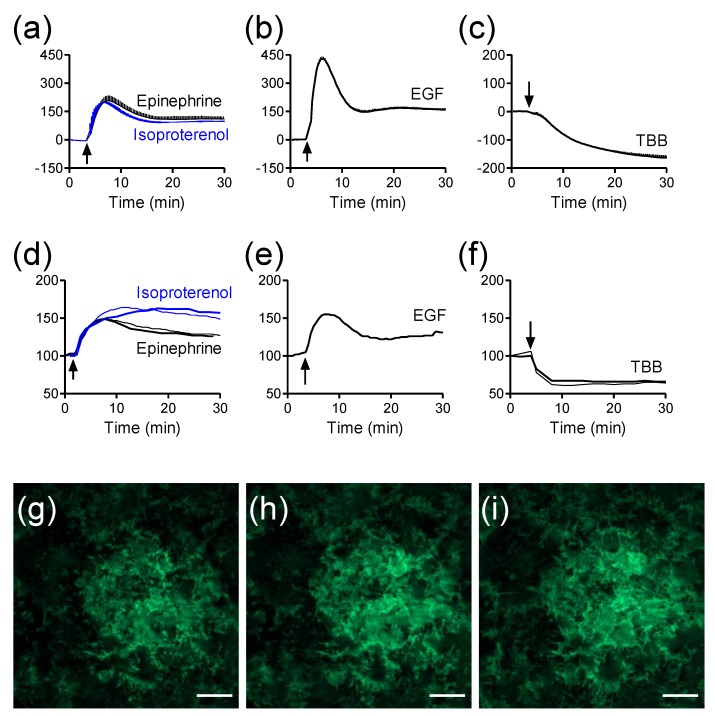
The label-free and TIRF profiles of receptor activation in HEK293-β2AR-GFP cells. (**a**–**c**) Real-time DMR signals induced by 10 µM epinephrine and 10 µM isoproterenol (**a**); 32nM EGF (**b**); and 10 µM TBB (**c**); (**d**–**f**) Real-time TIRF signals induced by 10 µM epinephrine and 10 µM isoproterenol (**d**); 32nM EGF (**e**); and 10 µM TBB (**f**); (**g**–**i**) TIRF images before (**g**), and 2 min (h) and 10 min (i) after the stimulation with 10 µM epinephrine. The data in (**a**–**c**) shows mean ± s.d. of eight replicates. Scale bar in **g**–**i** is 10 µm. This figure is reproduced with permission from Ref. [25].

Given that optical biosensors and TIRF all utilize surface-bound evanescent wave for cell assays, it is reasonable to hypothesize that TIRF can also be used for drug pharmacology profiling in a manner complementary to label-free biosensors. Using an engineered HEK293 cell line that stably expresses β_2_-AR with green fluorescent protein (GFP) at its C-terminal (β_2_AR-GFP) as the model, we compared the label-free DMR signals arising from the activation of several receptors with their respective TIRF signals [25]. The fluorescence intensity of β_2_AR-GFP was measured using a conventional TIRFM (Nikon Instruments Inc., Melville, NY, USA) and used as a cell surface fluorescence reporter for receptor activation. The DMR profiles of agonists were obtained using Epic^®^ BT system, a whole microplate-based RWG biosensor imager (Corning Incorporated, Corning, NY, USA) [32]. Results showed that the treatment with several ligands all triggered robust DMR and TIRF signals, but with distinct kinetics and amplitudes (Figure 5a–f). The two β_2_AR agonists epinephrine and isoproterenol triggered a transit positive DMR (Figure 5a), while epidermal growth factor (EGF), an agonist for the EGF receptor, also triggered a transit positive DMR but with much greater amplitude (Figure 5b). In contrast, the multi-kinase inhibitor TBB (4,5,6,7-tetrabromobenzotriazole) led to a sustained negative DMR (Figure 5c). Consistent with the fact that both label-free DMR and TIRF use surface bound evanescent wave for detection, we found that the signature of every ligand-induced TIRF kinetic profile obtained closely resembles its corresponding DMR signal (comparing Figure 4d with 4a for the two β_2_AR agonists, comparing Figure 4e with Figure 4b for EGF, and comparing Figure 4f with Figure 4c for TBB). Interestingly, the DMR and TIRF signatures of each agonist also display distinct fine features. This is because the RWG biosensor is mostly sensitive to dynamic redistribution of cellular components within the penetration depth (~150 nm) [75], while TIRF is a direct function of the distances of fluorescent receptors at the cell surface but within 100nm near the glass surface [1]. TIRF only detects the fluorescent receptors located at the cell surface membrane near the substrate surface (Figure 1g–i). Collectively, these results suggest that the TIRF measurements can provide useful information for assessing receptor pharmacology.

Combining label-free with TIRFM results can offer confirmative insights into receptor signaling. For instance, combining RWG-enabled DMR profiling with TIRF imaging can be used to ascertain the impact of receptor signaling on cell adhesion [24]. Results showed that β_2_-AR agonists, but not its antagonists or partial agonists, were capable of triggering signaling during the adhesion process, leading to an increase in the adhesion of HEK293-β_2_AR-GFP cells onto fibronectin-coated surfaces. In another study, Chabot *et al.* combined SPR-enhanced fluorescence (SPEF) microscopy with label-free SPR measurements to identify the molecular mechanisms responsible for the label-free profile of receptor signaling [77]. Results showed that in the actin filament labeled HEK293 cells angiotensin-II resulted in a biphasic SPR signal consisting of an initial decrease and a subsequent increase, which was correlated well with a biphasic SPEF micrograph showing a decrease in cellular area followed by actin densification and cell spreading.

Recently, Höglinger and colleagues used a conventional TIRFM to quantify glucose transporter-4 (GLUT4) translocation and to identify insulin mimetic drugs [78]. GLUT4 is an insulin-regulated glucose transporter found primarily in adipose tissues and striated muscle, as well as in the central nervous system, such as the hippocampus. The translocation of GLUT4 to the plasma membrane is critical to the insulin-stimulated transport of glucose in target tissues, and insulin resistance results in a decreased GLUT4 translocation efficiency. Using a highly insulin-sensitive CHO-K1 cell line that expresses a GLUT4-myc-GFP fusion protein they found that there is a positive correlation between increased TIRF signal and elevated glucose uptake induced by known insulin mimetic drugs. In another study, they combined micro-patterned surfaces with TIRFM to measure the interaction of growth factor receptor-bound protein 2 (Grb2) with EGFR [79]. Results showed that EGF stimulated the recruitment of Grb2 to the EGFR, which was significantly inhibited by known EGFR inhibitors, suggesting that TIRFM has a potential in profiling EGFR inhibitors.

## 6. Conclusions

TIRFM is a powerful tool to visualize cell plasma membrane associated events. However, TIRFM has found little use in high content screening up to now. With the development of a microplate compatible TIRF setup it has become clear that TIRFM can offer a unique means with moderate throughput to screen molecules that interact with cell membrane associated targets and cellular events. With the ability to control the penetration depth of the evanescent wave using different incident angles TIRFM system can also allow for probing multiple events occurring at different locations inside the cells. These developments pave a new way to screen and profile drugs.
